# Dynamic SARS-CoV-2-Specific Immunity in Critically Ill Patients With Hypertension

**DOI:** 10.3389/fimmu.2020.596684

**Published:** 2020-12-10

**Authors:** Qiang Zeng, Yong-Zhe Li, Sheng-Yong Dong, Zong-Tao Chen, Xiang-Yang Gao, Han Zhang, Gang Huang, Yang Xu

**Affiliations:** ^1^ Health Management Institute, The Second Medical Center & National Clinical Research Center for Geriatric Diseases, Chinese PLA General Hospital, Beijing, China; ^2^ Department of Laboratory Medicine, Peking Union Medical College Hospital, Beijing, China; ^3^ Health Management Center, The First Affiliated Hospital, Army Medical University, Chongqing, China; ^4^ Health Management Center, Beijing Aerospace General Hospital, Beijing, China; ^5^ Shanghai Key Laboratory of Molecular Imaging, Shanghai University of Medicine and Health Sciences, Shanghai, China

**Keywords:** COVID-19, SARS-CoV-2, hypertension, T cell, CD4, CD8

## Abstract

**Background:**

The current outbreak of coronavirus disease 2019 (COVID-19) caused by severe acute respiratory syndrome coronavirus 2 (SARS-CoV-2) poses an unprecedented health crisis. The most common chronic illness among patients infected with SARS-CoV-2 is hypertension. Immune dysregulation plays an important role in SARS-CoV-2 infection and in the development of hypertension; however, the dynamic immunological characteristics of COVID-19 patients with hypertension remain largely unclear.

**Methods:**

In total, 258 hypertensive patients infected with SARS-CoV-2 were included in this study. CD38^+^HLA-DR^+^ and CD38^+^PD-1^+^ CD8^+^ T cells, IFNγ^+^CD4^+^ and IFNγ^+^CD8^+^ T cells, the titers of IgG, IgM, and IgA against SARS-CoV-2 spike protein, and SARS-CoV-2 throat viral loads were measured weekly over 4 weeks after the onset of symptoms. Clinical outcomes were also monitored.

**Findings:**

CD4^+^ T lymphopenia was observed in 100% of the severe and critical cases. Compared with the surviving patients, the patients with fatal outcomes exhibited high and prolonged expression of CD38^+^HLA-DR^+^ and CD38^+^PD-1^+^ on CD8^+^ T cells, low expression of SARS-CoV-2-specific IFNγ^+^CD4^+^ and IFNγ^+^CD8^+^ T cells, low titers of IgG, IgM, and IgA against SARS-CoV-2 spike protein, and high SARS-CoV-2 viral load during the illness. In the surviving patients, the viral load was significantly inversely correlated with SARS-CoV-2-specific IFNγ^+^CD8^+^and IFNγ^+^CD4^+^ T cells, IgG, IgM, and IgA.

**Interpretation:**

T lymphopenia is common in critical or severe COVID-19 cases with hypertension. Prolonged activation and exhaustion of CD8^+^ T cells were associated with severe disease. The delayed SARS-CoV-2-specific antibody responses may be insufficient for overcoming severe SARS-CoV-2 infection in the absence of SARS-CoV-2-specific cellular responses.

## Introduction

Coronavirus disease 2019 (COVID-19) is currently a worldwide emergency. As of October 3, 2020, more than 1 million deaths and 34 million cases of severe acute respiratory syndrome coronavirus 2 (SARS-CoV-2) infection have been reported worldwide (1). T cells play a fundamental role in viral infections (2). It has been shown that SARS-CoV-2 infection causes overall reduction in the CD4^+^ and CD8^+^ T cell counts in peripheral blood (3, 4). The extent of this lymphopenia was correlated with COVID-19-associated disease severity and mortality (2, 5–8). Currently, little is known regarding the phenotype and function of T cell subsets in COVID-19. Generally, CD8^+^ T cells seem to be activated more than the CD4^+^ T cells (9, 10). The levels of programmed cell death protein 1 (PD-1) tend to increase in severe cases compared to those in the non-severe cases (3). Impaired cellular functionality in CD4^+^ and CD8^+^ T cells has been observed in severe COVID-19 cases along with generally lower interferon γ (IFNγ) and tumor necrosis factor α (TNF-α) production (11, 12). Furthermore, SARS-CoV-2 elicits a robust B cell response. Virus-specific IgM, IgG, and IgA have been detected in the days following infection (13). Additionally, high virus-specific antibody titers to SARS-CoV-2 have been observed to be inversely correlated with viral load in patients (14, 15). During the recovery stage of COVID-19 patients, T and B cells are highly expressed (16). In a recent study, SARS-CoV-2-specific T cells were detected in antibody-seronegative exposed family members and in convalescent asymptomatic and mild COVID-19 cases, suggesting that SARS-CoV-2 infection produces robust, broad, and highly functional memory T cell responses (17).

Research on SARS-CoV-2 infection has revealed that nearly half of the patients have a comorbidity, with hypertension being the most common (14). Generally, 30–35% of the COVID-19 patients have had a history of hypertension (14, 18). This rate reached up to 63% in critically ill patients (19). Compared to the patients without hypertension, those with hypertension had double the mortality risk (20). Similar to SARS-CoV-2 infection, the development of hypertension involves inflammation of the arterial wall. Interactions among the factors such as high blood pressure, pro-inflammatory cytokines, and T effector and regulatory lymphocytes play a critical role in the progression of hypertension (21–23). Among the patients infected with SARS-CoV-2, significant differences have been observed in immune cell counts between those with hypertension and those without hypertension (24). It is possible that the condition of the imbalanced immune system caused by hypertension may be exacerbated by SARS-CoV-2 infection, leading to a worse prognosis. Studies on the dynamic immunological characteristics of COVID-19 may improve the interpretation of disease pathogenesis and help to identify the potential factors involved in the treatment of the hypertension after COVID-19.

To the best of our knowledge, SARS-CoV-2-specific immunity in hypertensive patients, especially in critical cases, has not yet been reported. Here, we analyzed dynamic immunological characteristics in cases of survival versus cases of fatal COVID-19 patients with hypertension in a unique longitudinal cohort of samples. We aimed to identify the key differences in the immune responses after infection with SARS-CoV-2 between hypertensive patients who recovered and those who died.

## Materials and Methods

### Study Design and Participants

This retrospective study was conducted at Chinese PLA General Hospital, Peking Union Medical College Hospital, the First Affiliated Hospital, and the affiliated hospitals of Shanghai University of Medicine and Health Sciences. The study included 152 male and 106 female hypertensive patients {38.0% >65 years, with a mean age of 51 [standard deviation (SD) = 21)] and mean hypertension history of 16 [SD = 12] years} whose COVID-19 statuses were confirmed by SARS-CoV-2 nucleic acid assays. The 258 patients with COVID-19 were treated with thiazide diuretics (25.2%) and angiotensin-converting enzyme (ACE) inhibitors (74.8%). These hypertensive patients with COVID-19 were admitted from February 7, 2020 to March 1, 2020 (**Scheme S1**). This case series was approved by the institutional ethics board of Chinese PLA General Hospital (#2020-111), Peking Union Medical College Hospital (#ZS-1830), The First Affiliated Hospital (#KY2020046), and Shanghai University of Medicine and Health Sciences (#2019-LCHZ-18-20190507). Written informed consent was obtained from the controls and waived for the COVID-19 patients due to the rapid emergence of this infectious disease. Identification of hypertensive patients was achieved by reviewing and analyzing available electronic medical records and patient care resources. Clinical outcomes (discharge, mortality, and recovery, etc.) were monitored and all clinical recovery hypertensive patients meet the following criteria: body temperature returned to normal for more than 3 days, respiratory symptoms improved significantly, and lung imaging showed significant improvement.

Furthermore, due to a lack of reference ranges for lymphocyte and a subset profile of normal Chinese Han population, a total of 5,218 Chinese Han healthy individuals of 18-85 years obtained from clinic visits in November 2018 to November 2019, before the COVID-19 outbreak, were included and analyzed as controls.

### Cell Preparation

Whole blood samples were centrifuged for 15 min at 1,800 rpm to separate the cellular fraction and plasma. The plasma was then carefully removed and stored at -20°C. Peripheral blood mononuclear cells (PBMCs) were isolated using Ficoll-Paque PLUS density gradients (GE Healthcare Life Sciences, USA) according to the manufacturer’s instructions. Isolated PBMCs were either studied directly or cryopreserved in cell recovery media containing 10% DMSO (Gibco, USA), supplemented with 10% heat inactivated fetal bovine serum and stored in liquid nitrogen until used in the assays. Cryopreserved PBMCs were thawed by diluting them in 10 ml complete RPMI 1640 with 5% human AB serum (Gemini Bioproducts, USA) in the presence of benzonase (final concentration at 50 U/ml) before an experiment.

### SARS-CoV-2 Spike Glycoprotein Peptide Pools

SARS-CoV-2 Spike glycoprotein peptide pools (SPs,Cat. #RP30020) were obtained from Genscript Biotech, USA. The SPs included 316 peptides (delivered in two subpools of 158 peptides) derived from a peptide scan (15 mers with 11 amino acid overlap) of the entire Spike glycoprotein (Protein ID: P0DTC2) of SARS-CoV-2.

### Intracellular Cytokine Staining (ICS) Assay

PBMCs were first stimulated with or without SPs (2 μg/ml) for 3 h at 37°C, after which brefeldin A (10 μg/ml, Sigma-Aldrich, USA) was added to the cultures to enable intracellular proteins to accumulate in all stimulations. After incubation for a total of 6 h, the cells were washed, fixed, permeabilized using a fixation/permeabilization solution kit (BD Biosciences, USA) and blocked with Fc receptor (FcR) blocking reagent (Miltenyi Biotec, USA) for 30 min at 4°C to reduce nonspecific binding of antibodies to human Fc receptor. The cells were then stained with anti-IFNγ antibodies (BD Bioscience, USA) for 30 min at 4°C. After staining, all samples were washed twice with phosphate buffered saline (PBS) containing 0.1 % saponin, 0.1 % BSA and 0.05 % NaN_3_ (Sigma-Aldrich, USA), and resuspended in 300 μl PBS for measurement in a flow cytometer (FACSVerse*™* flow cytometry, BD Bioscience, USA).

### Real-Time Reverse Transcription Polymerase Chain Reaction (RT-PCR) Assay for SARS-CoV-2

Throat and blood samples were collected from hypertensive patients suspected of SARS-CoV-2 infection for SARS-CoV-2 RNA extraction. In brief, RNA was extracted within 2 h of collection using the total RNA isolation kit. Cell lysates (250 μl) were transferred into a collection tube and vortexed for 10 s. After standing at room temperature for 10 min, the collection tube was centrifugated at 1,000 rpm for 5 min. The suspension was then used for the RT-PCR assay for SARS-CoV-2 RNA. Two target genes, including open reading frame 1ab (ORF1ab) and nucleocapsid protein (N), were simultaneously amplified and tested during the RT-PCR assay. The primer and probe sequences were: Target 1 (ORF1ab): forward primer CCCTGTGGGTTTTACACTTAA; reverse primer ACGATTGTGCATCAGCTGA; and the probe 5′-VIC-CCGTCTGCGGTATGTGGAAAGGTTATGG-BHQ1-3′. Target 2 (N): forward primer GGGGAACTTCTCCTGCTAGAAT; reverse primer CAGACATTTTGCTCTCAAGCTG; and the probe 5′-FAM-TTGCTGCTGCTTGACAGATT-TAMRA-3′. The RT-PCR assay was performed using a SARS-CoV-2 nucleic acid detection kit. Reaction mixture contained 12 μl of reaction buffer, 4 μl of enzyme solution, 4 μl of probe primer solution, 3 μl of diethyl pyrocarbonate–treated water, and 2 μl of RNA template. The RT-PCR assay was performed under the following conditions: initial incubation at 50°C for 15 min followed by 95°C for 5 min, then 40 cycles of denaturation at 94°C for 15 s, and extension and collection off fluorescence signals at 55°C for 45 s. A cycle threshold value (C_t_-value) less than 37 was defined as a positive test result, and a C_t_-value of 40 or more was defined as a negative test. These diagnostic criteria were based on the recommendation by the National Institute for Viral Disease Control and Prevention (China). Samples with a C_t_-value of 37 to less than 40 was confirmed by retesting. The copies of RNA per reaction were obtained from the standard curve of limiting-dilution series of standard copies of RNA versus PCR amplification cycle.

### Enzyme-Linked Immunosorbent Assay (ELISA) of Immunoglobulin (Ig)

Antibodies (IgM, IgA, and IgG) specific to SARS-CoV-2 were determined using two different ELISA: an in-house assay that use SARS-CoV-2 Receptor Binding Domain (RBD) protein (Cat. #Z03479, Genscript Biotech, USA) as an antigen, and a commercial kit (SARS-CoV-2 Spike RBD ELISA Kit, Cat. #40591-V08H, Sino Biological, China). Microtiter plates were coated with 50 ng/well of target protein overnight at 4°C. Plates were then blocked for 2 h at 37°C using 200 μl of 5% non-fat milk in PBS. Serum samples were then diluted 1:50 using PBS and 100 μl of each sample was applied to the coated ELISA plate and incubated for 2 h at 37°C. Plates were then washed with PBS and incubated with HRP-labeled anti-human IgM, IgA, and IgG (Sigma Aldrich, USA), which were diluted to 1:2,000 in 5% non-fat milk in PBS. After incubation for another 1h at room temperature, the plates were washed and developed with TMB/E substrate (Millipore, USA). Finally, the reaction was stopped with 1 M H_2_SO_4_, and the optical density (OD) at 450 nm was measured. A negative serum control was run each time with the assay. A sample is positive if its adjusted OD value (OD of test – OD of control) exceeds the mean plus 3 standard deviations of the normal controls.

### Flow Cytometry Analysis

All samples were analyzed by FACSVerse*™* flow cytometry (BD Bioscience, USA). Two blood samples (100 μl each) were stained according to the manufacturer’s instructions. Then, red-cell lysis buffer (1 ml) was added to each sample and incubated for 10 min followed by washing with Sorvall cell washer (Thermo Fisher Scientific, USA). Cells were blocked with FcR blocking reagent (Miltenyi Biotec, USA) for 30 min at 4°C to reduce nonspecific binding of antibodies to human FcR and then washed with PBS. The cells were then stained with antibodies using the fixable dead cell stain kit (Invitrogen, USA) for 30 min at 4°C and were then resuspended in 350 μl PBS and analyzed in **a** flow cytometry (FACSVerse*™* flow cytometry, BD Bioscience, USA). Calibration and quality control for the instrument were carried out daily with the use of eight-color setup beads (BD Bioscience). All specimens were analyzed in duplicates with a coefficient of variation (CV) <5% by two independent technicians under the inter-laboratory quality control. The experiments were repeated if the results exhibited a CV >5% according to the instructions of BD Bioscience. The data were analyzed by FlowJo software (version 10, Tree Star, USA).

### Statistical Analysis

Categorical variables were described as frequency rates and percentages, and continuous variables were described using mean and median values. Means for continuous variables were compared using independent group *t* tests when the data were normally distributed; otherwise, the Mann-Whitney test was used. Data (non-normal distribution) from repeated measures were compared using Wilcoxon signed rank test and the generalized linear mixed model. Proportions for categorical variables were compared using the χ^2^ test, although the Fisher’s exact test was used when the data were limited. For unadjusted comparisons, a two-sided *P* value less than 0.05 was considered statistically significant. The analyses have not been adjusted for multiple comparisons, and given the potential for type I error, the findings should be interpreted as exploratory and descriptive. Because the cohort of hypertensive patients in our study were not derived from random selection, all statistics are deemed to be descriptive only. All statistical analyses were performed using SPSS (Statistical Package for the Social Sciences Inc., version 13.0).

## Results

### Dynamic Changes of CD4^+^ and CD8^+^ Lymphocyte Subsets in Peripheral Blood

The means of CD4^+^ and CD8^+^ T cells in the 5,218 hypertensive patients without COVID-19 were 872 (SD = 311) and 483 (SD = 216) cells/μl (**Table S1**). Among the 258 hypertensive patients with COVID-19, 207 treated with antiviral agents or corticosteroid therapy were excluded, thus 51 patients were included in our final analysis. Among them, 34 were mild cases and 17 were severe or critical cases. Eight critical cases died at week 5 or 6 (**Scheme S1**).

**Figure 1:**
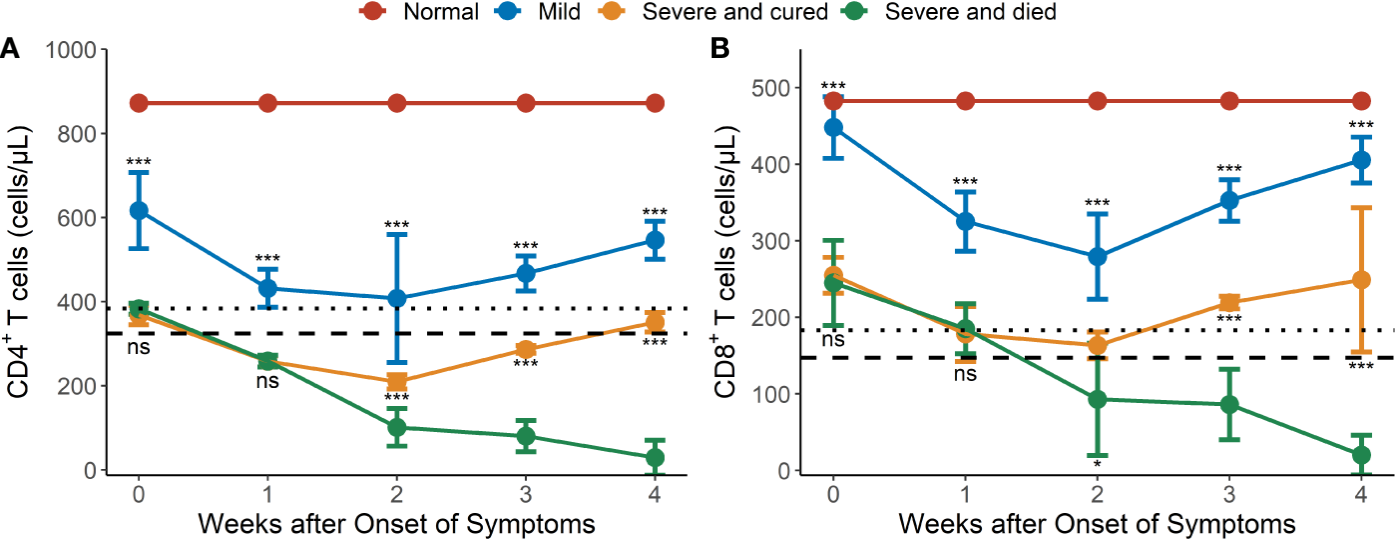
Dynamic changes of CD4 and CD8 lymphocyte subsets in peripheral blood of hypertensive patients with COVID-19. **(A)** Dynamic profile of CD4^+^ T cells in weeks 1-4. **(B)** Dynamic profile of CD8^+^ T cells in weeks 1-4. The means of CD4 and CD8 in normal controls were 872 and 483 cells/μl (redline). Dotted line: 2.5th percentile reference interval (RI); dash line: the 1st percentile RI. Error bars, mean ± SD. Differences between the mild cases (n = 34) and the severe or critical cases (n = 17) and between the surviving (n = 9) and fatal cases (n = 8) at any time point were compared with the Mann-Whitney test. ^*^ *P* < 0.05, ^***^ *P* < 0.001. ns, not significant.

The mean CD4^+^ and CD8^+^ T cell counts were significantly higher in mild cases than in severe or critical cases during the whole observation period (*P <*0.001 for all) (**Figures 1A, B**). Most of the hypertensive patients had reduced CD4^+^ and CD8^+^ T cell counts during the early phase of illness, which reached a trough in weeks 1–2 and recovered gradually afterwards, except for the fatal cases (**Figures 1A, B**). In the severe or critical cases, the levels of CD4^+^ and CD8^+^ T cell counts were not significantly different between those who survived and those who died during the early phase (weeks 1–2) of illness but after this it started to gradually increase in the survivors and continually decreased in the fatal cases. Differences in CD4^+^ and CD8^+^ T cell counts between the survival and fatal cases became significant between 2 to 4 weeks after COVID-19 onset (*P <*0.05 for all) (**Figures 1A, B**).

CD4^+^ T lymphopenia (mean CD4^+^ counts < 384 cells/μl) was observed in 100% of the severe or critical cases of COVID-19 in weeks 1–4 (**Figure 1A**) compared to the lower limit of normal controls (RI_2.5_ = 384 cells/μl, **Table S1**). However, CD8^+^ T lymphopenia (mean CD8^+^ counts < 183 cells/μl) was only observed in severe and died cases from 2 weeks after COVID-19 onset (**Figure 1B**) compared to the lower limit of the normal controls (RI_2.5_ = 183 cells/μl, **Table S1**).

### Prolonged Activation and Exhaustion of T Cells in Severe and Critical Cases of COVID-19

**Figure 2:**
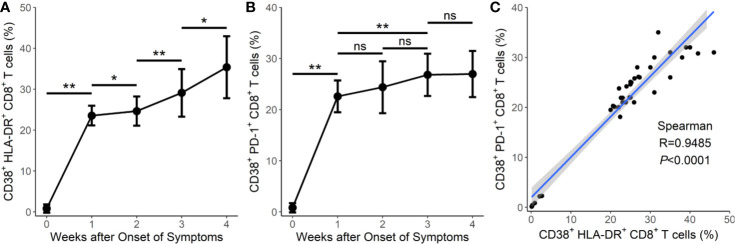
Prolonged activation and exhaustion in fatal cases of COVID-19. **(A)** Prolonged expression of CD38^+^HLA-DR^+^CD8^+^ T cells in the 8 fatal cases in weeks 1–4. **(B)** Prolonged expression of CD38^+^PD-1^+^CD8^+^ T cells in the 8 fatal cases in weeks 1–4. **(C)** Correlation between the frequency of CD38^+^HLA-DR^+^CD8^+^ T cells and the frequency of CD38^+^PD-1^+^CD8^+^ T cells in the 8 fatal cases. Error bars, mean ± SD. Statistical comparisons among time points were performed with the Wilcoxon signed rank test. ^*^ *P* < 0.05, ^**^ *P* < 0.01. ns, not significant.

We then tested the expression of CD38 and HLA-DR, key markers of CD8^+^ T cell activation, as well as PD-1, an activation and exhaustion marker of CD8^+^ T cells, during SARS-CoV-2 infection. Our results showed that the CD38^+^HLA-DR^+^ expression on CD8^+^ T cells in the surviving hypertensive patients was transiently high. Early in the infection at week 1, the frequency of CD38^+^HLA-DR^+^CD8^+^ T cells in the survival group reached a mean of 29.4%, then gradually declined to 4.53% as the hypertensive patients clinically recovered at week 4 (**Table 1**). Conversely, the fatal cases exhibited a high and prolonged expression of CD38^+^HLA-DR^+^ on their CD8^+^ T cells, starting with a mean of 23.6% at week 1 and reaching 35.3% at week 4 (**Table 1**, **Figure 2A**). These differences in the proportion of activated CD38^+^HLA-DR^+^CD8^+^ T cells in weeks 3–4 were significant between the fatal hypertensive patients and the survivors (*P <*0.05 for all) (**Table 1**).

**Table 1:** Prolonged and dysregulated expression of CD38^+^HLA-DR^+^ and CD38^+^PD-1^+^ on CD8^+^ T cells in the fatal and surviving cases.

Time	Week 1		Week 2		Week 3		Week 4	
CD8^+^ subsets	CD38^+^HLA-DR^+^	CD38^+^PD-1^+^	CD38^+^HLA-DR^+^	CD38^+^PD-1^+^	CD38^+^HLA-DR^+^	CD38^+^PD-1^+^	CD38^+^HLA-DR^+^	CD38^+^PD-1^+^
Fatal (%), n = 8	23.6 ± 2.41	22.6 ± 3.11	24.7 ± 3.56	24.4 ± 5.06	29.1 ± 5.81	26.8 ± 4.13	35.3 ± 7.57	26.9 ± 4.51
Survival (%), n = 9	29.4 ± 7.79	17.8 ± 6.41	19.3 ± 7.34	15.1 ± 6.29	9.08 ± 3.21	4.61 ± 1.09	4.53 ± 0.96	2.08 ± 0.66
*P*	0.900	0.506	0.482	0.679	<0.05	<0.05	<0.001	<0.001

Similar results were obtained for CD38^+^PD-1^+^CD8^+^ T cells (**Table 1**, **Figure 2B**). The proportion of CD38^+^PD-1^+^CD8^+^ T cells was significantly higher in the fatal cases of COVID-19 than those found in the survivors 3 weeks later (P <0.05 for all) (**Table 1**). In addition, there was a significant correlation between the CD38^+^HLA-DR^+^ and CD38^+^PD-1^+^ CD8^+^ T cell subsets in the fatal cases (**Figure 2C**; R = 0.9485, *P* < 0.0001 by Spearman correlation test).

### SARS-CoV-2-Specific Cellular and Humoral Immunity in Severe and Critical Cases of COVID-19

**Figure 3:**
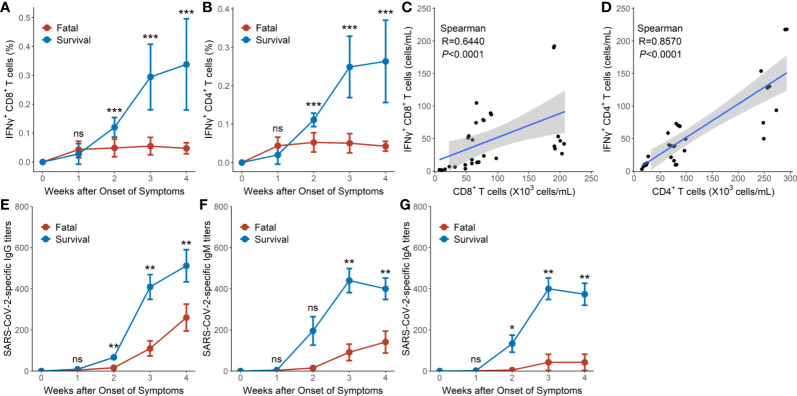
SARS-CoV-2-specific cellular and humoral immunity in hypertensive patients with COVID-19. **(A)** Dynamic profile of IFNγ^+^CD8^+^ T cells of the 9 surviving and 8 fatal cases in weeks 1–4. **(B)** Dynamic profile of IFNγ^+^CD4^+^ T cells of the 9 surviving and 8 fatal cases in weeks 1–4. **(C)** Correlation between the frequency of IFNγ^+^CD8^+^ T cells and CD8^+^ T cells in the 8 fatal cases. **(D)** Correlation between the frequency of IFNγ^+^CD4^+^ T cells and CD4^+^ T cells in the and 8 fatal cases. **(E)** Dynamic profile of IgG against SARS-CoV-2 spike protein in the 9 surviving and 8 fatal cases in weeks 1–4. **(F)** Dynamic profile of IgM against SARS-CoV-2 spike protein in the 9 surviving and 8 fatal cases in weeks 1–4. **(G)** Dynamic profile of IgA against SARS-CoV-2 spike protein in the 9 surviving and 8 fatal cases in weeks 1–4. Error bars, mean ± SD **(A, B)** or mean ± standard error **(E–G)**. Differences between the survival cases and the fatal cases at any time point were compared with the Mann-Whitney test. ^*^ *P* < 0.05, ^**^ *P* < 0.01, ^***^ *P* < 0.001. ns, not significant.

Early in the infection at week 1, the proportions of SARS-CoV-2-specific IFNγ^+^CD4^+^ and IFNγ^+^CD8^+^ T cells in the survival group reached mean values of 0.021 and 0.027%, respectively, then gradually increased to 0.263 and 0.337%, respectively, as hypertensive patients clinically recovered at week 4 (**Table 2**, **Figures 3A, B**). Conversely, the fatal cases displayed prolonged low expressions of SARS-CoV-2-specific IFNγ^+^CD4^+^ and IFNγ^+^CD8^+^ T cells (**Figures 3A, B**). Thus, the proportion of SARS-CoV-2-specific IFNγ^+^CD4^+^ and IFNγ^+^CD8^+^ T cells was significantly lower in the fatal cases when compared to the survivors 2 weeks later (*P <*0.001 for all) (**Table 2**, **Figures 3A, B**).

**Table 2:** Loss expansion of SARS-CoV-2-specific IFNγ^+^CD4^+^ and IFNγ^+^CD8^+^ on T cells in the fatal and surviving cases.

Time	Week 1		Week 2		Week 3		Week 4	
Virus-specific T cells	IFNγ^+^CD4^+^	IFNγ^+^CD8^+^	IFNγ^+^CD4^+^	IFNγ^+^CD8^+^	IFNγ^+^CD4^+^	IFNγ^+^CD8^+^	IFNγ^+^CD4^+^	IFNγ^+^CD8^+^
Fatal (%), n = 8	0.043 ± 0.022	0.043 ± 0.028	0.0525 ± 0.024	0.048 ± 0.030	0.0514 ± 0.025	0.055 ± 0.030	0.043 ± 0.013	0.047 ± 0.019
Survival (%), n = 9	0.021 ± 0.025	0.027 ± 0.035	0.111 ± 0.018	0.120 ± 0.033	0.249 ± 0.081	0.294 ± 0.113	0.263 ± 0.107	0.337 ± 0.157
*P*	0.056	0.335	<0.0001	0.0004	<0.0001	<0.0001	<0.0001	0.00012

Significant correlations were, however, observed between SARS-CoV-2-specific IFNγ^+^CD8^+^T cell numbers and CD8^+^ T cell counts (R = 0.6440, *P* < 0.0001 by Spearman correlation test) as well as between SARS-CoV-2-specific IFNγ^+^CD4^+^ T cell numbers and CD4^+^ T cell counts (R = 0.8570, *P* < 0.0001 by Spearman correlation test) in the fatal cases (**Figures 3C, D**).

The titers of IgG, IgM, and IgA against SARS-CoV-2 spike protein in the survival group reached mean titers of 67, 196, and 133 at week 2, respectively, then gradually increased to mean titers of 538, 398, and 373, respectively, as the hypertensive patients clinically recovered at week 4 (**Figures 3E–G**). Conversely, hypertensive patients with fatal disease outcomes displayed relatively low titers of IgG, IgM, and IgA, starting with a mean titer of 16, 15, and 5, respectively, at week 2 and reaching mean titers of 260, 141, and 43 respectively at week 4 (**Figures 3E–G**). Thus, the proportions of IgG, IgM, and IgA titers were significantly lower in the fatal hypertensive patients than in the survivors at 2 or 3 weeks later (*P <*0.05 for all) (**Figures 3E–G**).

### SARS-CoV-2-Specific T Cells Correlate With SARS-CoV-2-Specific Ig

Analysis of SARS-CoV-2-specific IFNγ^+^CD8^+^ and IFNγ^+^CD4^+^ T cells, and SARS-CoV-2-specific Ig of the surviving hypertensive patients revealed that anti-SARS-CoV-2-spike protein IgG significantly correlated with the frequency of SARS-CoV-2-specific IFNγ^+^CD8^+^ T cells (R = 0.8647, *P* < 0.0001) and the frequency of SARS-CoV-2-specific IFNγ^+^CD4^+^ T cells (R = 0.9171, *P* < 0.0001) (**Figures S1A, S1B**).

### SARS-CoV-2-Specific Immunity Correlates With Virus Clearance

**Figure 4:**
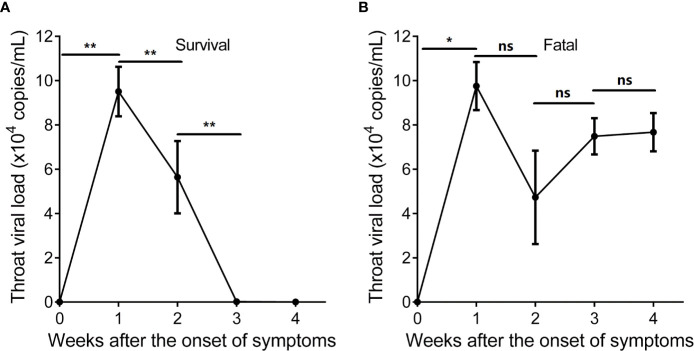
Longitudinal profile of SARS-CoV-2 viral load of hypertensive patients with COVID-19 in weeks 1–4 after the onset of symptoms. **(A)** Longitudinal profile of SARS-CoV-2 viral load of the 9 surviving hypertensive patients in weeks 1–4. **(B)** Longitudinal profile of SARS-CoV-2 viral load of the 8 fatal hypertensive patients in weeks 1–4. Error bars, mean ± SD. Statistical comparisons among time points were performed with the Wilcoxon signed rank test. ^*^ *P* < 0.05, ^**^ *P* < 0.01. ns, not significant.

We investigated the SARS-CoV-2-specific immunity at time points with detectable SARS-CoV-2 throat viral load. The SARS-CoV-2 viral load of the surviving hypertensive patients peaked at week 1 and tested negative in weeks 3–4 (**Figure 4A**). Conversely, the profile of SARS-CoV-2 viral load showed persistently positive tests for 4 weeks in the fatal cases (**Figure 4B**).

Analysis of IFNγ^+^CD8^+^ or IFNγ^+^CD4^+^ T cells and SARS-CoV-2 viral load of the surviving hypertensive patients revealed that the SARS-CoV-2 viral load significantly inversely correlated with the frequency of IFNγ^+^CD8^+^T cells (R = -0.8636, *P* < 0.0001) or IFNγ^+^CD4^+^ T cells (R = - 0.8543, *P* < 0.0001) (**Figures S2A, S2C**).

Analysis of IFNγ^+^CD8^+^ or IFNγ^+^CD4^+^ T cells and SARS-CoV-2 viral load of the 8 fatal hypertensive patients revealed that SARS-CoV-2 viral load did not or weakly correlated with the frequency of IFNγ^+^CD8^+^ T cells (R = -0.3941, *P* = 0.0256) or IFNγ^+^CD4^+^ T cells (R = - 0.1969, *P* = 0.2802) (**Figures S2B, S2D**).

Analysis of SARS-CoV-2-specific IgG, IgM, IgA, and SARS-CoV-2 viral load of the surviving hypertensive patients revealed that SARS-CoV-2 viral load significantly inversely correlated with the titers of SARS-CoV-2-specific IgG (R = -0.8962, *P* < 0.0001), IgM (R = -0.6735, *P* < 0.0001), and IgA (R = -0.6916, *P* < 0.0001) (**Figure S3**).

## Discussion

Our study analyzed dynamic immunological characteristics in a cohort of 51 hypertensive patients with COVID-19. T lymphopenia was the most common finding in our study. Furthermore, both CD38^+^HLA-DR^+^CD8^+^ T cells and CD38^+^PD-1^+^CD8^+^ T cells were decreased in severe and cured patients and increased in severe and died patients within weeks after onset of symptoms. Conversely, the proportions of IFNγ^+^CD4^+^ and IFNγ^+^CD8^+^ T cells increased with weeks after onset of symptoms in severe and cured patients but had prolonged low expression in severe and died patients. Moreover, the titers of IgG, IgM, and IgA against SARS-CoV-2 spike protein, also within weeks after onset of symptoms, increased in severe and cured patients compared with the fatal cases. Finally, the profile of SARS-CoV-2 viral load tested negative in weeks 3–4 in the surviving cases but were persistently positive for the 4 weeks in the fatal cases. SARS-CoV-2 viral load significantly inversely correlated with the frequency of IFNγ^+^CD8^+^ T cells and SARS-CoV-2-specific IgG, IgM, and IgA in the surviving cases but not in the fatal cases. These findings suggest that T cells are critical to the clinical outcomes in hypertensive patients with COVID-19. Compared with the severe and cured patients, the severe and died patients exhibited immune loss expansion of SARS-CoV-2-specific cellular and humoral immunity resulting in prolonged SARS-CoV-2 exposure.

Levels of lymphocytes and lymphocyte subsets have great importance in keeping the immune system working. Usually viral infection, immunodeficiency diseases, and other infectious diseases lead to abnormal changes in the levels of lymphocyte subsets (2). A number of studies have reported that the disease severity of COVID-19 is associated with lymphopenia (8, 25, 26). We observed that CD4^+^ and CD8^+^ lymphocytes were above the normal controls in mild cases during illness, were under the normal controls at admission but then returned to normal range in severe but cured cases, and continuously declined, reaching close to zero, in the severe and died cases. This suggests that lymphocytes are critical to clinical outcomes. Although the extent of lymphopenia in COVID-19 patients correlates with COVID-19 severity and mortality, the mechanism by which SARS-CoV-2 impacts the human immune system is still unclear (2).

CD38 and HLA-DR are key markers of CD8^+^ T cell activation (27). Day et al. (28) showed that disease severity, as determined by the viral load and the declining CD4^+^ counts, correlated with both the PD-1 expression levels on HIV-specific CD8^+^ T cells and the proportion of cells expressing PD-1, thus providing a marker on CD8^+^ T cells for disease severity. PD-1 expression levels were also associated with decreased virus-specific CD8^+^ T cell proliferation in response to *in vitro* stimulation with the HIV antigen. Collectively, these findings show that the level of PD-1 correlates with the extent of T cell exhaustion. It has been reported that the disease severity of COVID-19 is associated with dysregulation of CD38^+^, HLA-DR^+^, or PD-1^+^ CD8^+^ T cells (3, 29). Compared with the survivors, our data showed that the fatal cases of COVID-19 exhibited high and persistent expression of CD38^+^HLA-DR^+^ and CD38^+^PD-1^+^on CD8^+^ T cells as well as high levels of SARS-CoV-2 throat viral load, which may be indicative of prolonged virus exposure in the fatal cases. We provide the evidence that prolonged activation and exhausted CD8^+^ T cells are associated with severe disease in the hypertensive patients who succumbed to SARS-CoV-2 infection.

After the SARS outbreak in 2003 and the H1N1 pandemic in 2009, determining whether patients have long-term immunity after infection has become a major concern (30–34). Channappanavar et al. (35) first reported that SARS-CoV-specific memory CD8^+^ T cells persisted for up to 6 years after SARS-CoV infection. When challenged with a lethal dose of SARS-CoV, virus-specific memory CD8^+^ T cells efficiently produced IFNγ, TNF-α, etc. and reduced the lung viral load. Our data showed that the proportions of IFNγ^+^CD4^+^ and IFNγ^+^CD8^+^ T cells increased and inversely correlated with SARS-CoV-2 viral load in surviving hypertensive patients with severe COVID-19 but not in the fatal cases. Furthermore, Ni et al. (5) reported SARS-CoV-2-specific humoral and cellular immunity in 14 convalescent patients and showed that neutralizing antibody titers correlated with titers of the IgG antibody against the Spike protein receptor-binding domain (RBD) of SARS-CoV-2. However, we observed that SARS-CoV-2-specific humoral immunity, such as IgG, IgM, and IgA against the RBD of SARS-CoV-2, was recovered after the expansion of SARS-CoV-2-specific T cells and positively correlated with the frequency of IFNγ^+^CD4^+^ and IFNγ^+^CD8^+^ T cells in surviving cases. These findings indicate that the surviving hypertensive patients with severe COVID-19 have an appropriate expansion of SARS-CoV-2 specific cellular and humoral immunity that can clear the virus. Meanwhile, it appears that in the absence of SARS-CoV-2-specific cellular responses, the delayed SARS-CoV-2-specific antibody responses may be insufficient for overcoming severe COVID-19.

Importantly, the magnitude of SARS-CoV-2-specific T cells in peripheral blood represents the active migration of SARS-CoV-2-specific T cells from lymph nodes to the infection sites and might correlate with the magnitude of SARS-CoV-2-specific T cell response in the lungs and in the lymph nodes. Our data indicated that CD4^+^ and CD8^+^ T cells in the peripheral blood early after infection might be a good predictor of prognosis and thereby lead to appropriate alternative treatment strategies for COVID-19 due to the significantly high correlation of CD4^+^ and CD8^+^ T cells with SARS-CoV-2-specific IFNγ^+^CD4^+^ and IFNγ^+^CD8^+^ T cells, respectively.

This study has several limitations. First, although our data exhibits the dynamic immune responses as the disease progresses in hypertensive patients with COVID-19. We did not compare differences in the dynamic immune responses between the COVID-19 patients with hypertension and those without hypertension. Thus, although the literature reported that immune dysregulation was more severe in hypertensive patients compared with the patients without hypertension (24), we cannot conclude that the higher mortality risks can be attributed to the severity of immune dysregulation in hypertensive patients with COVID-19. Second, the analyses have not been adjusted for multivariate factors, such as age, gender, and medication use, because of the small size of the cohort of hypertensive patients in our study. A number of studies have reported the effect of ACE inhibitors on COVID-19 prognosis (36, 37).

In summary, we found that the extent of lymphopenia in COVID-19 patients with hypertension correlates with COVID-19 severity and mortality. The early prominence of activated CD38^+^HLA-DR^+^ and CD38^+^PD-1^+^ CD8^+^ T cells is associated with survival; the loss of expansion of SARS-CoV-2-specific immunity and the prolonged presence of CD38^+^HLA-DR^+^ and CD38^+^PD-1^+^ CD8^+^ T cells are characteristics of fatal COVID-19. The proportions of IFNγ^+^CD4^+^ and IFNγ^+^CD8^+^ T cells and the titers of IgG, IgM, and IgA against SARS-CoV-2 spike protein increased and inversely correlated with the SARS-Cov-2 viral load in surviving hypertensive patients with severe COVID-19. These findings suggest that T lymphopenia is common in critical or severe COVID-19 patients with hypertension. Furthermore, prolonged activation and exhaustion of CD8^+^ T cells were associated with severe disease. Meanwhile, T cells were shown to be critical to clinical outcomes. It appears that in the absence of SARS-CoV-2-specific cellular responses, the delayed SARS-CoV-2-specific antibody responses may be insufficient for overcoming severe COVID-19. Our findings provided a basis for further analysis of SARS-CoV-2-specific immunity and provides further understanding of the pathogenesis of hypertension followed by COVID-19.

## Data Availability Statement

The raw data supporting the conclusions of this article will be made available by the authors, without undue reservation.

## Ethics Statement

The studies involving human participants were reviewed and approved by the institutional ethics board of Chinese PLA General Hospital (#2020-111), Peking Union Medical College Hospital (#ZS-1830), The First Affiliated Hospital (#KY2020046), and Shanghai University of Medicine and Health Sciences (#2019-LCHZ-18-20190507). The patients/participants provided their written informed consent to participate in this study. Written informed consent was obtained from the individual(s) for the publication of any potentially identifiable images or data included in this article.

## Author Contributions

QZ and Y-ZL contributed to study design, data interpretation, and manuscript drafting. S-YD contributed to data analysis, figure preparation, the literature search, and manuscript drafting. Z-TC, X-YG, and HZ contributed to performed experiments and data collection. GH and YX contributed to study design and reviewed the final draft. All authors contributed to the article and approved the submitted version.

## Funding

The work was supported in part by the National Natural Science Foundation of China (Grant No. 81830052 and 81530053). The funders had no role in study design, data collection and analysis, decision to publish, or preparation of the manuscript.

## Conflict of Interest

The authors declare that the research was conducted in the absence of any commercial or financial relationships that could be construed as potential conflict of interest.
