# Supplementary material for: Dynamic SARS-CoV-2-Specific Immunity in Critically Ill Patients With Hypertension
Source: Front Immunol. 2020 Dec 10;11:596684. doi: 10.3389/fimmu.2020.596684 (PMC7758245; doi:10.3389/fimmu.2020.596684)
Supplement: Supplementary file 1 [file DataSheet_1.docx]

***Supplemental material***

**1 Tables**

**Table S1. Lymphocyte and subset profile of the 5,218 controls**

| **Contents***  **（n=5,218）** | **Median** | **RI （2.5-95.5%tile）** | **RI （1-99%tile）** | **Mean** | **SD** |
| --- | --- | --- | --- | --- | --- |
| Lymphocytes (cells/μL) | 1,940 | 1,060-3,271 | 928-3,659 | 2,013 | 776 |
| Lymphocytes (%) | 33.1 | 18.7-47.9 | 15.7-50.5 | 33.3 | 7.5 |
| CD3^+^ (cells/μL) | 1,394 | 719-2,463 | 611-2,737 | 1,448 | 454 |
| CD4^+^ (cells/μL) | 832 | 384-1,580 | 324-1,810 | 872 | 311 |
| CD8^+^ (cells/μL) | 448 | 183-976 | 147-1,174 | 483 | 216 |
| CD4^+^/CD8^+^ (ratio) | 1.86 | 0.77-4.34 | 0.64-5.09 | 2.05 | 0.94 |
| CD19^+^ (cells/μL) | 235 | 75-586 | 51-669 | 268 | 549 |
| CD16^+^CD56^+^ (cells/μL) | 218 | 67-643 | 46-810 | 255 | 160 |

*Due to a lack of reference ranges for the lymphocyte and subset profile of the normal Chinese Han population, we systematically analyzed 5,218 healthy Chinese individuals, aged 18-85 years, from clinic visits between November 2018 and November 2019, before the COVID-19 outbreak, as controls. The above analyses have not been adjusted for multivariate factors, such as age, gender, and medication use.

**Table S2. Characteristics of age and sex in the 5,218 controls**

|  | Total (n=5,218) | Male (n=4,044) | Women (n=1,174) | *P* value |
| --- | --- | --- | --- | --- |
| Age (year) | 52 (47, 57) | 51 (46, 55) | 55 (51, 62) | <0.0001 |

Data are shown as median (interquartile range).

**2. Figures**


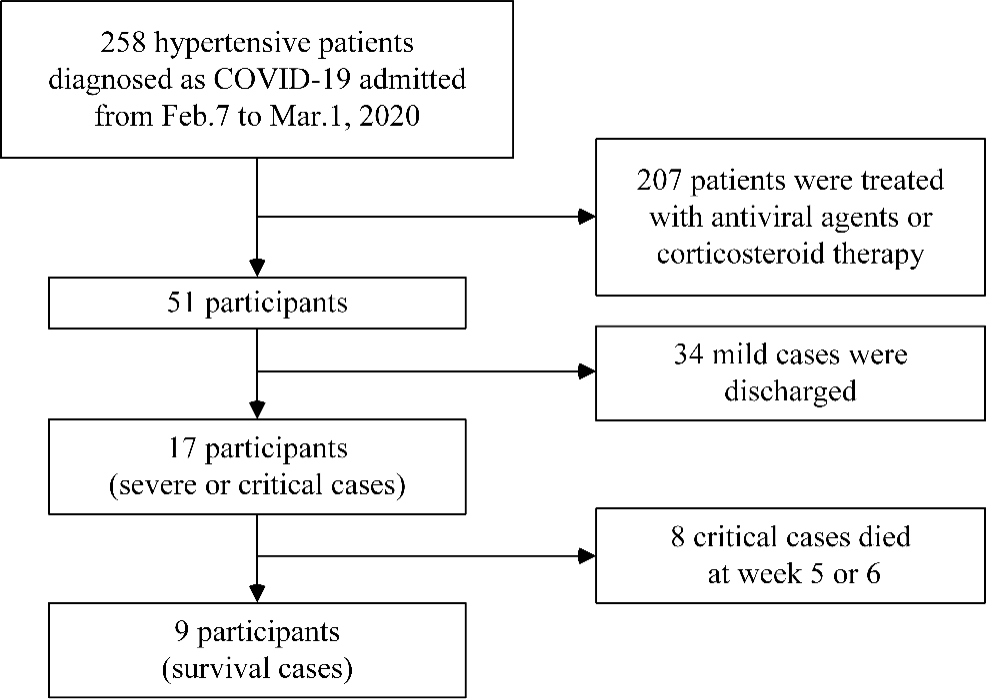


**Scheme S1. A schematic overview illustrating participant enrollment.**


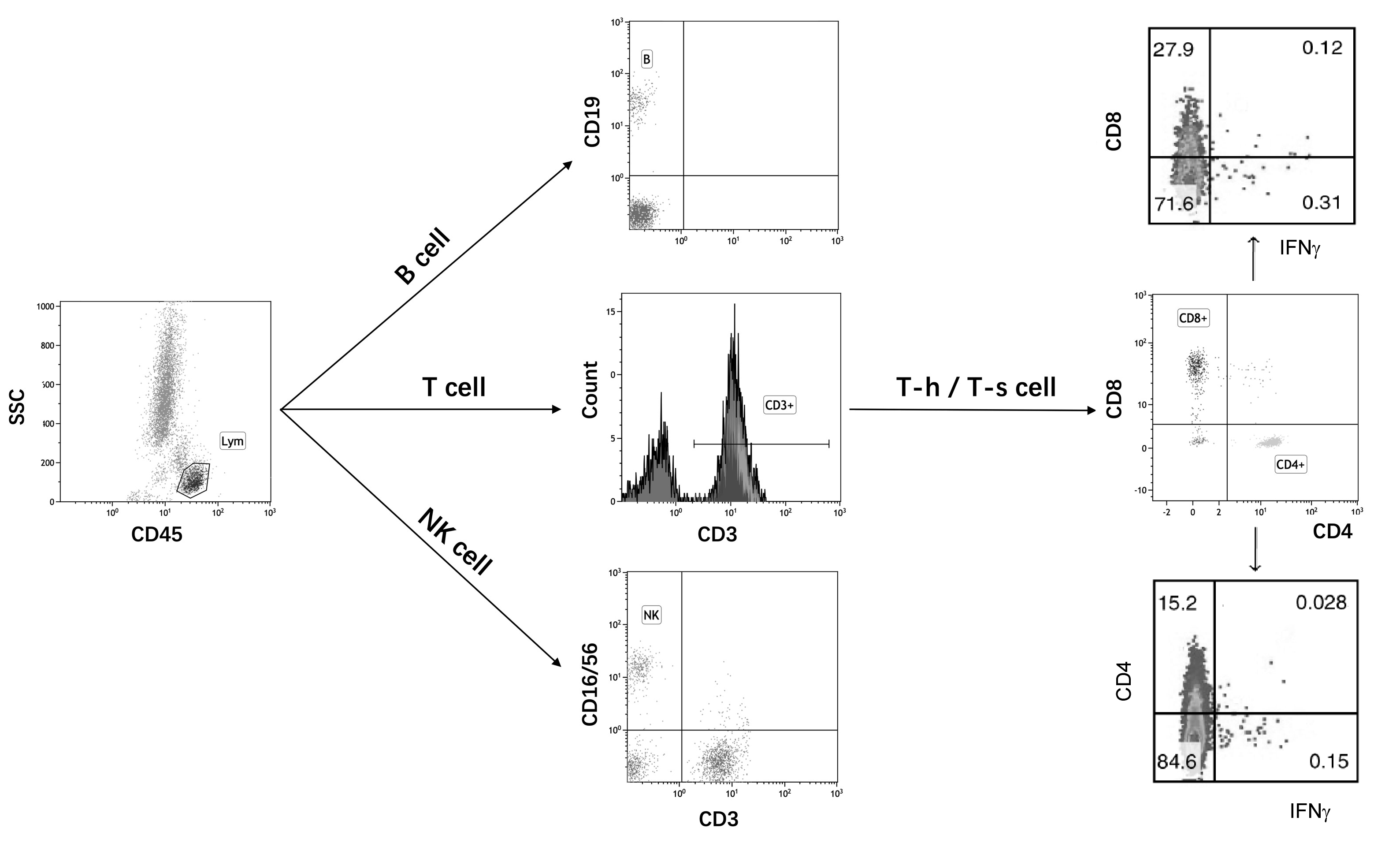


**Scheme S2. A representative flow cytometry gating strategy.**

**
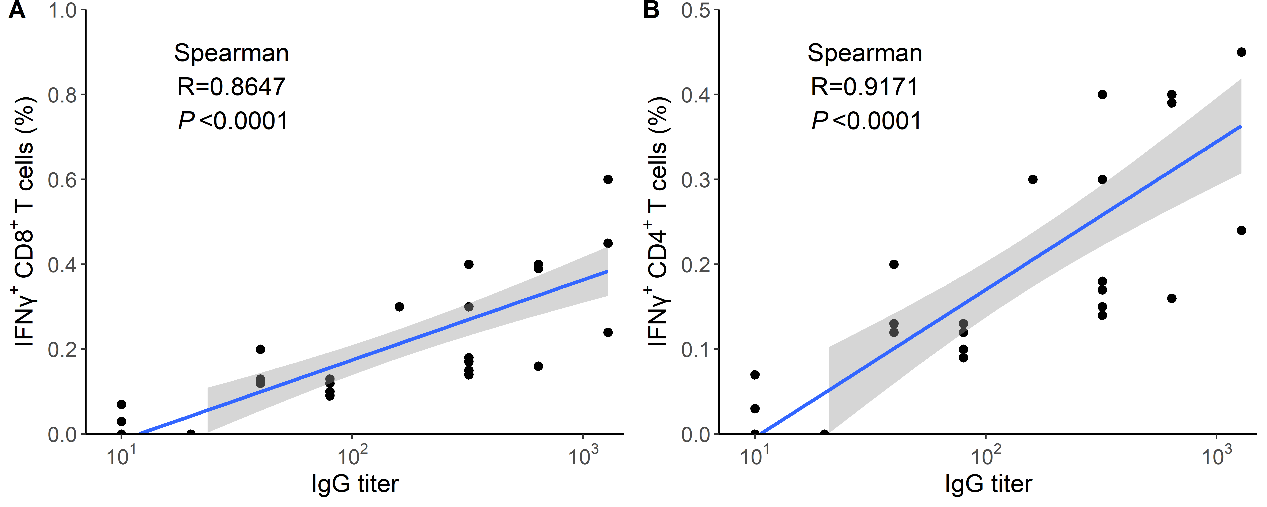
**

**Figure S1. SARS-CoV-2-specific IFNγ^+^CD8^+^and IFNγ^+^CD4^+^T cells correlated with the SARS-CoV-2-specific immunoglobulin G.** (A) Correlation between SARS-CoV-2-specific IFNγ^+^CD8^+^ T cells (%) and anti-SARS-CoV-2 IgG titers (R = 0.8647, *P* < 0.0001) of the nine surviving hypertensive patients in weeks 1-4. (B) Correlation between SARS-CoV-2-specific IFNγ^+^CD4^+^T cells (%) and anti-SARS-CoV-2 IgG titers (R = 0.9171, *P* < 0.0001) of the nine surviving hypertensive patients in weeks 1-4. Data were obtained from nine hypertensive patients over four weeks, and tests were performed weekly. Statistical comparisons were performed using the Spearman correlation test.


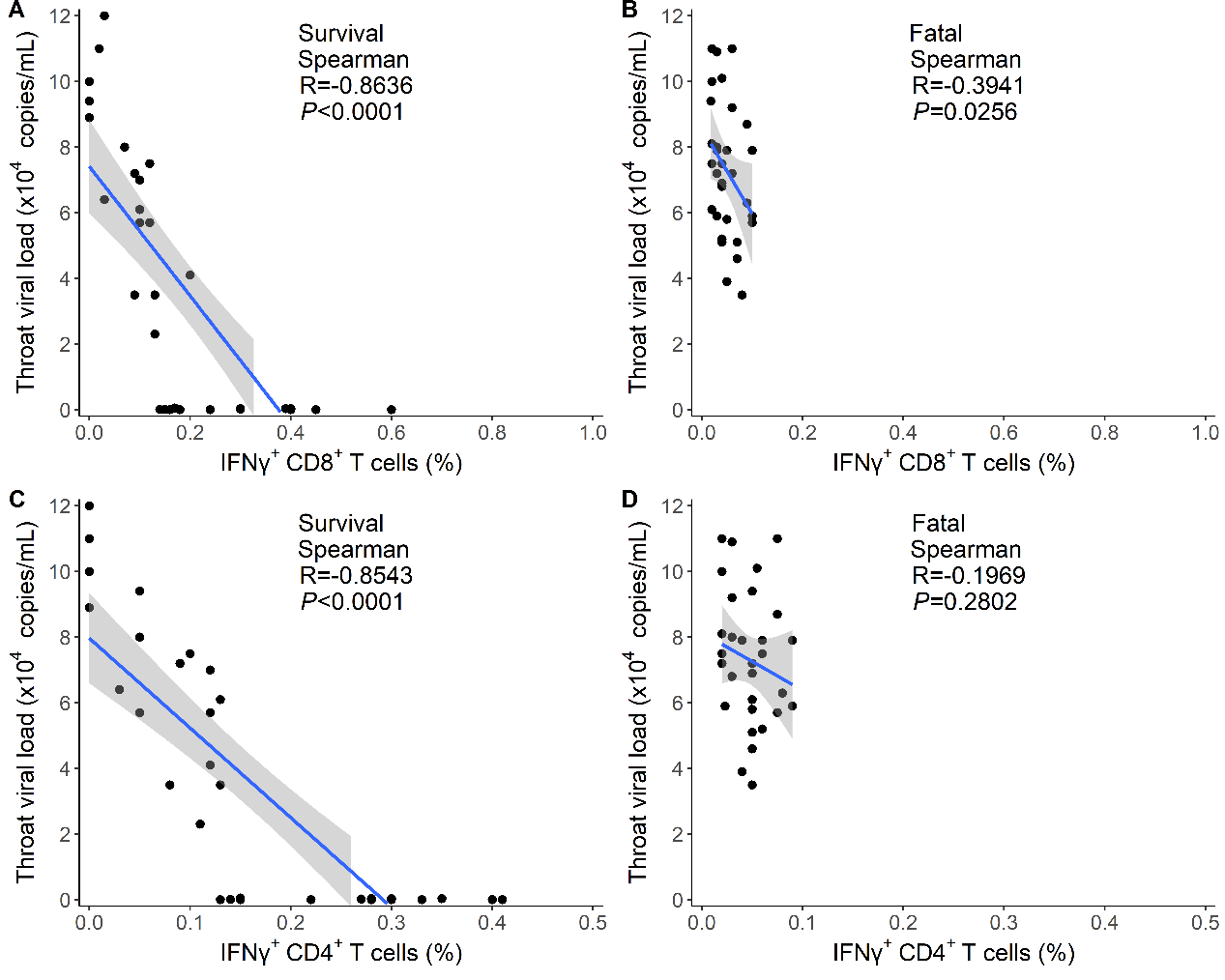


**Figure S2. IFNγ^+^CD8^+^ or IFNγ^+^CD4^+^ T cells correlated with the SARS-CoV-2 viral load of hypertensive patients with COVID-19 in weeks 1-4.** (A) Inverse correlation of SARS-CoV-2-specific IFNγ^+^CD8^+^ T cells vs. the SARS-CoV-2 viral load is shown (R = -0.8636, *P* < 0.0001) in the nine surviving cases in weeks 1-4. (B) Weak inverse correlation of SARS-CoV-2-specific IFNγ^+^CD8^+^T cells vs. the SARS-CoV-2 viral load is shown (R = -0.3941, *P* = 0.0256) in the eight fatal cases in weeks 1-4. (C) Inverse correlation of SARS-CoV-2-specific IFNγ^+^CD4^+^ T cells vs. the SARS-CoV-2 viral load is shown (R = - 0.8543, *P* < 0.0001) in the nine surviving cases in weeks 1-4. (D) Weak inverse correlation of SARS-CoV-2-specific IFNγ^+^CD4^+^T cells vs. the SARS-CoV-2 viral load is shown (R = - 0.1969, *P* = 0.2802) in the eight fatal cases in weeks 1-4. Data were obtained from nine surviving cases or eight fatal cases and tests were done weekly. Statistical comparisons were performed using the Spearman correlation test.


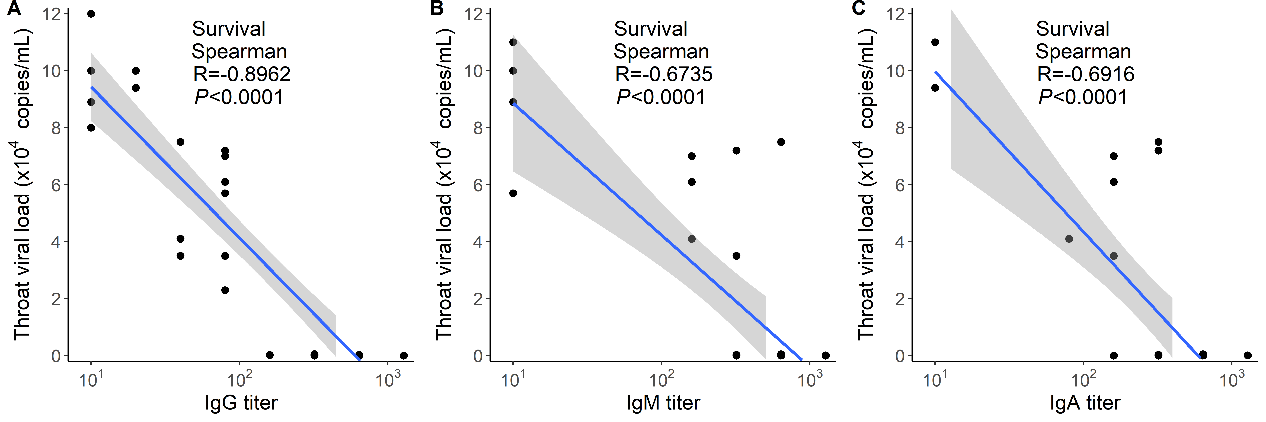


**Figure S3. SARS-CoV-2-specific IgG, IgM, and IgA correlated with the SARS-CoV-2 viral load of the nine surviving hypertensive patients with COVID-19.** (A) Inverse correlation between SARS-CoV-2-specific IgG titers and the SARS-CoV-2 viral load (R = -0.8962, *P* < 0.0001) in weeks 1-4. (B) Inverse correlation between SARS-CoV-2-specific IgM titers and the SARS-CoV-2 viral load (R = -0.6735, *P* < 0.0001) in weeks 1-4. (C) Inverse correlation between SARS-CoV-2-specific IgA titers and the SARS-CoV-2 viral load (R = -0.6916, *P* < 0.0001) in weeks 1-4.Data were obtained from nine surviving hypertensive patients over four weeks, and tests were done weekly. Statistical comparisons were performed using the Spearman correlation test.

KEY RESOURCES TABLE

| **REAGENT or RESOURCE** | **SOURCE** | **IDENTIFIER** |
| --- | --- | --- |
| **Antibodies** | | |
| CD3-FITC | BD Biosciences | Cat# 337166; BD Multitest 6-color TBNK reagent |
| CD16/56-PE | BD Biosciences | Cat# 337166; BD Multitest 6-color TBNK reagent |
| CD45-PerCP-Cy™5.5 | BD Biosciences | Cat# 337166; BD Multitest 6-color TBNK reagent |
| CD4-PE-Cy™7 | BD Biosciences | Cat# 337166; BD Multitest 6-color TBNK reagent |
| CD19-APC | BD Biosciences | Cat# 337166; BD Multitest 6-color TBNK reagent |
| CD8-APC-Cy7 | BD Biosciences | Cat# 337166; BD Multitest 6-color TBNK reagent |
| IFNγ-FITC | BD Biosciences | Cat#554700 |
| CD38-FITC | Beckman Coulter | Cat# IM0775U |
| HLA-DR-PE | Beckman Coulter | Cat# IM1639U |
| PD-1-PE | Beckman Coulter | Cat# B30634 |
|  |  |  |
| **Chemicals, Peptides, and Recombinant Proteins** | | |
| SARS-CoV-2 Spike glycoprotein peptide pools | Genscript Biotech | RP30020, |
| Ficoll-Paque PLUS | GE Healthcare Life Sciences, | 17144002 |
| Cell recovery media | GIBCO | 12648010 |
| Human AB serum | Gemini Bioproducts | 100-318 |
| Brefeldin A | Sigma-Aldrich | ab120299 |
| Saponin | Sigma-Aldrich | 8047-15-2 |
| SARS-CoV-2 receptor binding domain protein | Genscript Biotech | Z03479 |
| HRP-labeled anti-human Ig A, IgG, IgM, | Sigma Aldrich | AP120P |
| TMB/E substrate | Millipore | ES001 |
| **Critical Commercial Assays** | | |
| SARS-CoV-2 Spike RBD ELISA Kit | Sino Biological, China | 40591-V08H |
| **Software and Algorithms** | | |
| FlowJo 10 | FlowJo | https://www.flowjo.com/ |
| SPSS | IBM | <https://www.ibm.com/analytics/spss-statistics-software> |

**RESOURCE AVAILABILITY**

#### Lead Contact

Further information and requests for resources and reagents should be directed to and will be fulfilled by the Lead Contact,

#### Materials Availability

This study did not generate new unique reagents.

### Data and Code Availability

Data of this study are available after communication with the Lead Contact.
